# Evaluation of Alveolar Bone Height Changes Following Clear Aligner Therapy: A Retrospective Analysis Using Orthopantomograms

**DOI:** 10.7759/cureus.90858

**Published:** 2025-08-24

**Authors:** Shilpa Pharande, Parvesh Parvesh, Rajat Sharma, Chand Sawhney, Aminesh Bisht, Kailash Gorchhia, Seema Gupta

**Affiliations:** 1 Department of Orthodontics, Sinhgad Dental College and Hospital, Sinhgad, IND; 2 Department of Orthodontics, Jan Nayak Chaudhary Devilal Dental College, Sirsa, IND; 3 Department of Orthodontics, Maharaja Ganga Singh Dental College and Research Centre, Sriganganagar, IND; 4 Department of Orthodontics, Maharana Pratap College of Dentistry and Research Centre, Gwalior, IND; 5 Department of Orthodontics, Rama Dental College Hospital and Research Centre, Kanpur, IND; 6 Department of Orthodontics, Sanjeevani Hospital, Sirsa, IND; 7 Department of Orthodontics, Kothiwal Dental College and Research Centre, Moradabad, IND

**Keywords:** alveolar bone loss, clear aligners, orthodontic treatment, orthopantomogram, periodontal health

## Abstract

Introduction: The rising demand for clear aligners, attributed to their discreet appearance and removability, underscores the need to assess their effects on periodontal health, particularly alveolar bone height. This study aimed to investigate the changes in alveolar bone height in patients undergoing clear aligner therapy using pre- and post-treatment orthopantomograms (OPGs), contributing to the evidence for their clinical application.

Materials and methods: This retrospective study was conducted at Kothiwal Dental College and Research Centre (Moradabad, UP, IND) on OPG records of 50 periodontally healthy adults (aged 18 to 40 years) treated with clear aligners for non-extraction cases involving maxillary and/or mandibular expansion. Patients with systemic disease, periodontal disease, or prior orthodontic treatment were excluded. Pre- and post-treatment OPGs obtained using standardized settings were evaluated for alveolar bone height from the cementoenamel junction to the alveolar crest on the incisors, canines, and first molars. Two calibrated orthodontists performed blinded measurements with intraclass correlation coefficients ≥0.90, ensuring reliability. Data were analyzed using paired t-tests for pre- and post-treatment comparisons and multivariable linear regression to assess confounding factors (age, sex, and treatment duration). Statistical significance was set at p < 0.05.

Results: Significant changes in alveolar bone height were observed across all regions (p < 0.001), with the mandibular anterior region showing the greatest alveolar bone loss of 0.31 mm, followed by the mandibular posterior (-0.30 mm), maxillary anterior (-0.21 mm), and maxillary posterior regions (-0.07 mm). Class I and II malocclusions exhibited significant changes (-0.90 mm in class I and -0.86 mm in class II), whereas class III malocclusions were not significant (-0.34 mm, p = 0.078). Multivariable linear regression analysis revealed no significant associations between the examined parameters and overall alveolar bone level changes in patients undergoing clear aligner therapy (p > 0.05) for all variables except sex (p = 0.041) and treatment duration (p = 0.017), with relatively small effect sizes.

Conclusion: Clear aligner therapy induced minimal but significant changes in alveolar bone height, particularly in the mandibular region, supporting periodontal safety. These findings underscore the need for careful treatment planning and periodontal monitoring to optimize outcomes in modern orthodontics.

## Introduction

Orthodontic treatment has evolved significantly with the advent of clear aligners that offer an aesthetic and comfortable alternative to traditional fixed appliances. Clear aligners made of transparent thermoplastic materials facilitate tooth movement through a series of custom-designed trays that incrementally reposition the teeth [1]. Although their efficacy in achieving dental alignment is well-documented, their impact on supporting structures such as alveolar bone and tooth roots remains a critical area of investigation [2]. Understanding these effects is essential, as the health of periodontal tissues and root integrity directly influence long-term treatment outcomes and patient oral health.

Orthopantomograms (OPGs) and panoramic radiographs are widely used in orthodontics to assess dental and skeletal structures before and after treatment. They provide a comprehensive view of the maxilla, mandible, and associated structures, making them valuable for evaluating changes in alveolar bone height and root length [3]. The alveolar bone, which anchors the teeth within the jaws, is subject to remodeling during orthodontic tooth movement owing to the application of controlled forces. These forces stimulate osteoclastic and osteoblastic activity, potentially altering bone height and density [4]. Similarly, root resorption, a known risk factor for orthodontic treatment, may lead to reductions in root length, compromising tooth stability if severe [5]. Evaluating these changes in the context of clear aligners is particularly relevant, as the biomechanical properties of aligners differ from those of fixed appliances, potentially influencing periodontal and root responses [6].

Clear aligners exert lighter intermittent forces than the continuous forces of traditional braces, which may result in distinct patterns of bone remodeling and root resorption [6,7]. A previous systematic review has suggested that controlled and gradual force application of aligners may minimize adverse effects on periodontal tissues, but comprehensive evidence is limited [7]. Pre- and post-treatment OPGs allow clinicians to quantify changes in alveolar bone height, typically measured as the distance from the cementoenamel junction to the alveolar crest, and root length, assessed by comparing root dimensions relative to stable anatomical landmarks [6]. These measurements provide insight into the safety and biological impact of clear aligner therapy.

The growing popularity of clear aligners, driven by the patient demand for discrete and removable appliances, underscores the need to thoroughly evaluate their effects on periodontal health. Although aligners offer advantages such as improved oral hygiene and reduced soft tissue irritation, their influence on the alveolar bone and root structure must be critically assessed to ensure treatment safety [8]. Factors such as treatment duration, force magnitude, patient age, and periodontal status may modulate these outcomes, necessitating a detailed analysis of OPG findings. This study aimed to investigate the changes in alveolar bone height using pre- and post-treatment OPGs in patients undergoing clear aligner therapy, contributing to the evidence for their clinical application. By elucidating these effects, clinicians can predict treatment outcomes, optimize protocols, and enhance patient care in modern orthodontics.

## Materials and methods

This retrospective study was conducted at Kothiwal Dental College and Research Centre (Moradabad, UP, IND) using OPGs from a four-year period (December 2019 to December 2023). Ethical approval was obtained from the Institutional Ethics Review Board of Kothiwal Dental College (approval No. KDCRC/IERB/2/2024/O121), and the study adhered to the Declaration of Helsinki. Patient data were anonymized to ensure confidentiality, and informed consent was not required because of the retrospective nature of the study.

Eligible patients were adults aged 18 to 40 years who were systemically and periodontally healthy, as confirmed by medical history and periodontal examination, including a probing depth of ≤3 mm, no bleeding on probing, and no clinical attachment loss. Only patients treated with clear aligners (K Line Europe GmbH, Düsseldorf, DEU) for non-extraction with maxillary and/or mandibular expansion were included. Treatment addressed minor crowding, deep bite, or spacing, with aligners changed every two weeks, and treatment was completed within 12 to 15 months. Patients underwent oral prophylaxis using ultrasonic scalers (EMS Piezon; EMS Dental, Nyon, CHE) and polishing with Prophy-Mate (NSK-Nakanishi International, Tokyo, Japan) before treatment. Exclusion criteria included patients with systemic diseases, bone disorders, smokers, patients taking antibiotics or corticosteroids in the three months prior to the study, periodontal disease, previous orthodontic treatment, missing teeth, craniofacial anomalies, or poor-quality OPGs. All patients were treated by the same orthodontist, who has more than 15 years of experience. 

A total of 100 patient records were screened from the digital archive, and 50 records were selected based on the power analysis. The sample size was determined using G*Power software version 3.1.9.2 (Heinrich Heine University, Düsseldorf, DEU), by using two prior dependent means (paired t-test family) and based on an effect size of 0.35 (a study analyzed change in alveolar bone levels in aligner therapy), a 5% margin of error, and 80% statistical power, yielding a required sample of 50 patients [6]. Additionally, to assess intra-observer reliability, 20% of the total sample (10 patients) was evaluated.

**Figure 1:**
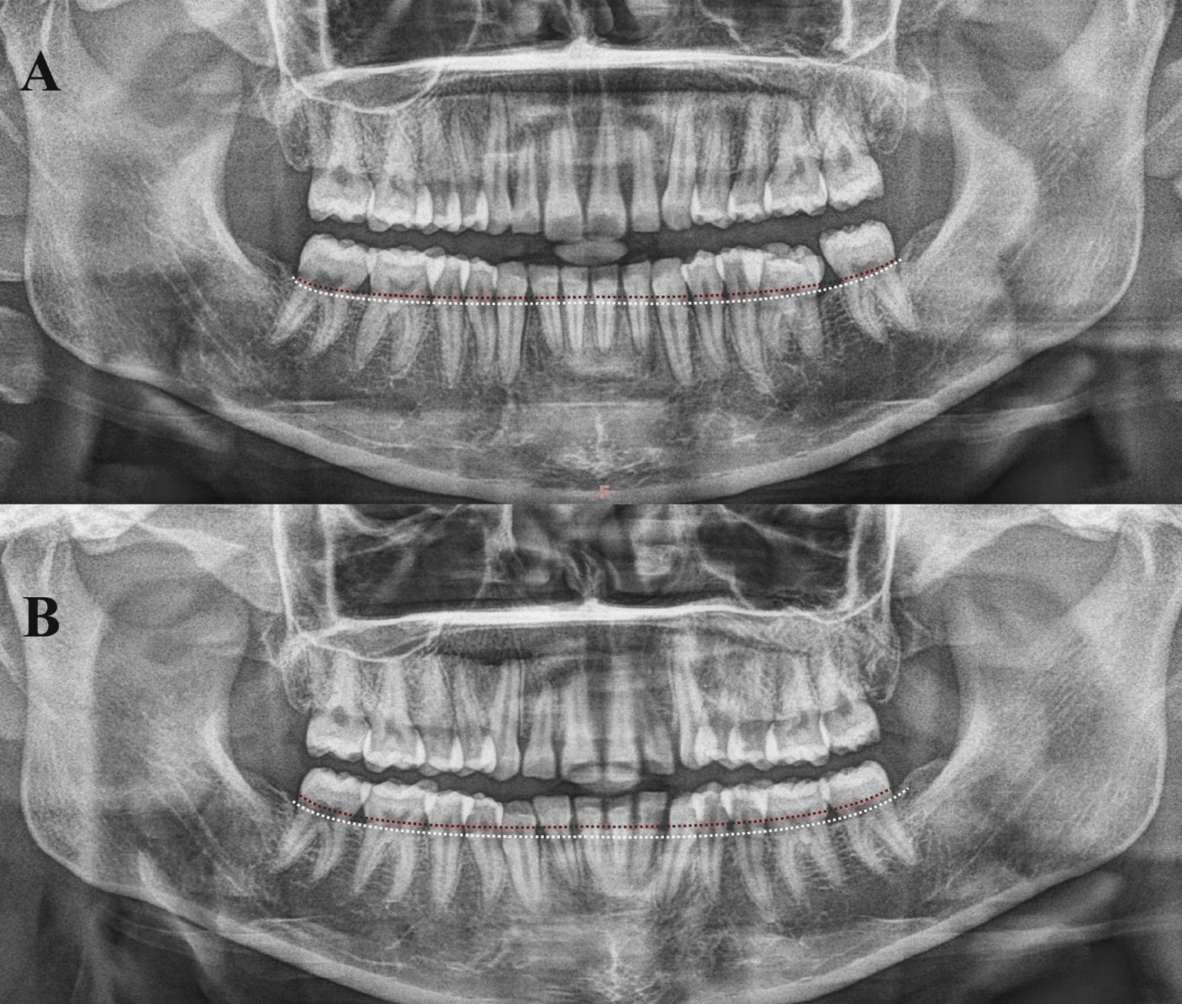
Panoramic radiograph of a patient pre- (A) and post-treatment with clear aligners These original OPG images of a patient from the study, used with permission, show the level of the alveolar bone crest (white interrupted line) and the level of the CEJ (red interrupted line) CEJ: Cementoenamel junction, OPG: Orthopantomogram

The OPGs were obtained (Planmeca ProMax 2D; Planmeca Oy, Helsinki, FIN) at standardized settings (70 kVp, 10 mA, 12 s exposure). Pre- and post-treatment OPGs were analyzed using Planmeca Romexis software (version 5.2, Planmeca Oy) to measure alveolar bone height (Figure 1). Alveolar bone height was measured as the distance from the CEJ to the alveolar crest on the mesial and distal aspects of the maxillary and mandibular incisors, canines, and first molars. The data about the patient encompassed various factors such as the patient's age, sex, classification of malocclusion (class I, class II, or class III), duration of orthodontic intervention (<1 or one to two years), whether interproximal stripping was conducted during the orthodontic process, and alterations in the alveolar bone level measured in millimeters.

Two calibrated orthodontists, authors Pharande and Sawhney, each with over five years of experience and from different colleges, independently performed the measurements while blinded to patient identity and treatment stage. Calibration involved training on 10 non-study OPGs, with intraclass correlation coefficients (ICC) calculated to ensure reliability (ICC ≥ 0.90 for all measurements). Discrepancies exceeding 0.5 mm were resolved by consensus. Intra- and inter-examiner reliabilities were assessed using 10 randomly selected OPGs, measured twice with a two-week interval (ICC ≥ 0.92).

Data were recorded in Microsoft Excel (Microsoft Corp., Redmond, WA, USA) and analyzed using SPSS Statistics version 20 (IBM Corp., Armonk, NY, USA) by a statistician (author Parvesh) who was provided with coded data. The normality test (Shapiro-Wilk test) confirmed a normal distribution, permitting parametric analysis. Descriptive statistics (frequencies, percentages, means, and standard deviations) were used to summarize the patient characteristics and pre- and post-treatment alveolar bone levels across the maxillary and mandibular teeth. Paired t-tests were used to compare pre- and post-treatment changes in alveolar bone height. Additionally, a multivariate linear regression analysis was performed to assess confounding factors influencing bone loss, adjusting for variables such as age, sex, and treatment duration. Statistical significance was set at p < 0.05.

## Results

The study population comprised 26 (52%) men and 24 (48%) women. Class I malocclusion was the most prevalent in 32 (64%) patients, followed by class II in 16 (32%), and class III in two (4%). Interproximal stripping was performed in 20 (40%) patients, whereas 30 (60%) patients did not require interproximal stripping. Most patients underwent treatment for more than one year. Maxillary arch expansion was performed in 11 (22%) patients, while mandibular expansion was performed in 20 (40%) patients (Table 1).

**Table 1:** Basic characteristics of study population undergoing clear aligner therapy Data are presented as frequency (n) and percentage (%), where n denotes number of patients.

Parameters	Category	Frequency (n)	Percentage
Sex	Male	26	52
	Female	24	48
Malocclusion	Class I	32	64
	Class II	16	32
	Class III	2	4
Interproximal stripping	No	30	60
	Yes	20	40
Duration of treatment	>1 year	38	76
	<1 year	12	24
Maxillary arch expansion	No	39	78
	Yes	11	22
Mandibular arch expansion	No	30	60
	Yes	20	40

The descriptive analysis revealed minimal pre-treatment differences in alveolar bone height between males (4.78 ± 0.22 mm) and females (4.73 ± 0.28 mm), with both showing comparable post-treatment change in bone levels. Class I malocclusion demonstrated the highest pre-treatment (4.80 ± 0.27 mm) and post-treatment (5.71 ± 0.20 mm) alveolar bone height values, while class III showed the least change in alveolar bone levels (4.84 ± 0.17 mm to 5.16 ± 0.19 mm). The interproximal stripping had a negligible impact. Similarly, the treatment duration showed consistent changes in bone levels (Table 2).

**Table 2:** Descriptive statistics of pre- and post-treatment alveolar bone height (in mm) across different parameters Data are presented as range, mean, and standard deviation.

Parameter	Category	Pre-treatment	Range	Post-treatment	Range
Sex	Male	4.78 ± 0.22	4.46-5.23	5.64 ± 0.22	5.38-6.12
	Female	4.73 ± 0.28	4.32-5.23	5.65 ± 0.21	5.28-5.92
Occlusion	Class 1	4.80 ± 0.27	4.32-5.23	5.71 ± 0.20	5.36-6.12
	Class 2	4.64 ± 0.17	4.38-4.86	5.50 ± 0.19	5.28-5.83
	Class 3	4.84 ± 0.17	4.81-4.90	5.16 ± 0.19	5.12-5.22
Stripping	No	4.77 ± 0.23	4.38-5.23	5.66 ± 0.19	5.28-5.97
	Yes	4.73 ± 0.28	4.32-5.23	5.63 ± 0.26	5.36-6.12
Duration	>1 year	4.75 ± 0.26	4.32-5.23	5.65 ± 0.23	5.28-6.12
	<1 year	4.76 ± 0.24	4.46-5.13	5.63 ± 0.18	5.38-5.82

A paired t-test analysis revealed a statistically significant (p < 0.001) change in alveolar bone levels across nearly all parameters following clear aligner therapy. Both sexes showed comparable mean differences (males: -0.86 mm; females: -0.92 mm). Class I (-0.90 mm) and class II (-0.86 mm) malocclusions demonstrated significant alveolar bone-level changes, whereas class III changes (-0.34 mm) were non-significant (p = 0.078). The interproximal stripping status (no: -0.89 mm; yes: -0.89 mm) and treatment duration (>1 year: -0.90 mm; <1 year: -0.87 mm) showed similar significant improvement. These results suggest that clear aligner therapy altered alveolar bone levels (Table 3).

**Table 3:** Comparison of pre- and post-treatment alveolar bone level changes (in mm) across different parameters in patients undergoing clear aligner therapy, using the paired t-test Mean difference (post-treatment measurements – pre-treatment measurements) in mm; *p < 0.05 denotes statistical significance

Parameters	Category	Mean difference (mm)	CI at 95%		t-value	p-value
			Lower limit	Upper limit		
Sex	Male	0.86	-0.94	-0.78	-22.19	0.001*
	Female	0.92	-0.99	-0.84	-25.82	0.001*
Malocclusion	Class I	0.90	-0.98	-0.83	-23.63	0.001*
	Class II	0.86	-0.93	-0.79	-27.06	0.001*
	Class III	0.34	-0.46	-0.25	-1.78	0.078
Interproximal stripping	No	0.89	0.95	-0.82	-28.53	0.001*
	Yes	0.89	-0.99	-0.79	-18.61	0.001*
Duration of treatment	>1 year	0.90	-0.95	-0.84	-32.07	0.001*
	<1 year	0.87	-1.02	-0.72	-12.74	0.001*

A paired t-test analysis revealed a statistically significant change (p < 0.001) in alveolar bone levels across all regions following clear aligner therapy. The mandibular anterior region showed the greatest mean difference (-0.31 mm), followed by the mandibular posterior (-0.30 mm) and maxillary anterior (-0.21 mm) regions. The maxillary posterior region demonstrated the smallest, yet significant improvement (-0.07 mm). These findings suggest that clear aligner therapy changed alveolar bone levels, with more pronounced effects observed in the mandibular region than in the maxillary region (Table 4).

**Table 4:** Comparison of pre- and post-treatment alveolar bone level changes in patients undergoing clear aligner therapy, using the paired t-test Data are presented as mean ± standard deviation (SD) and range in mm; *p < 0.05 denotes statistical significance; mean difference = post-treatment measurements – pre-treatment measurements

Region of measurement of alveolar bone levels	Pre-treatment alveolar bone levels (mm)		Post-treatment alveolar bone levels (mm)		Mean difference (mm)	t-value	p-value
	Mean ± Std.	Range	Mean ± Std.	Range			
Maxillary anterior	0.96 ± 0.11	0.78-1.12	1.17 ± 0.09	0.98-1.34	-0.21	-14.76	0.001*
Maxillary posterior	1.21 ± 0.15	0.97-1.46	1.27 ± 0.15	1.00-1.69	-0.07	-6.61	0.001*
Mandibular anterior	1.29 ± 0.11	1.09-1.48	1.60 ± 0.19	1.28-1.93	-0.31	-17.33	0.001*
Mandibular posterior	1.30 ± 0.10	1.12-1.467	1.60 ± 0.13	1.36-1.87	-0.30	-13.90	0.001*

Multivariable linear regression analysis revealed no significant associations between the examined parameters and overall alveolar bone level changes in patients undergoing clear aligner therapy (p > 0.05) for all variables except sex (p = 0.041) and treatment duration (p = 0.017), with relatively small effect sizes. These results suggest that alveolar bone-level changes during clear aligner therapy occur independently of these patient-specific and treatment-related factors, indicating that the therapeutic effects on the alveolar bone may be more universally applicable across different clinical scenarios (Table 5).

**Table 5:** Multivariable linear regression analysis of overall alveolar bone level changes in patients undergoing clear aligner therapy Unstandardized coefficient (B) represents alveolar bone level change in mm; *p < 0.05 denotes statistical significance

Parameters	Reference	Unstandardized coefficient (B)	Standard error	t-value	p-value
Sex (male)	Female	-0.064	0.065	-1.986	0.041*
Interproximal stripping (yes)	No	0.024	0.059	0.414	0.681
Duration of treatment (>1 year)	<1 year	-0.008	0.078	-2.104	0.017*
Maxillary jaw expansion (yes)	No	-0.084	0.068	-1.237	0.223
Mandibular jaw expansion (yes)	No	0.031	0.059	0.521	0.605
Malocclusion (class II)	Class I	-0.024	0.061	-0.395	0.695
Malocclusion (class III)	Class I	-0.133	0.175	-0.761	0.451

## Discussion

The findings of this retrospective study provided valuable insights into the effects of clear aligner therapy on alveolar bone height and contribute to the growing body of evidence on the periodontal implications of this increasingly popular orthodontic treatment. The statistically significant changes in alveolar bone levels observed across various parameters, particularly in the mandibular regions, underscore the potential of clear aligners to influence periodontal health outcomes.

These findings suggest that clear aligner therapy may induce favorable remodeling of the alveolar bone, particularly in the mandibular arch, which can be attributed to the biomechanical properties of the aligners and their controlled force application. For instance, previous studies have reported that clear aligners are better for periodontal health than fixed appliances in terms of plaque index, probing depth, and gingival index [7,9]. The present study did not evaluate the periodontal indices; therefore, we could not compare these findings with those of our study. Barreda et al. [10] reported a reduction in the alveolar bone levels on the buccal side of the maxillary first premolar region. In contrast, Figueiredo et al. [11] reported no significant change in alveolar bone thickness after maxillary arch expansion with Invisalign (Align Technology Inc., Tempe, AZ, USA) therapy. Alasqah et al. [6] reported similar findings with a significant reduction in alveolar bone levels in patients undergoing clear aligners.

The controlled and intermittent forces exerted by aligners, as opposed to the continuous forces of fixed appliances, may contribute to these outcomes by minimizing excessive stress on the periodontal ligament and alveolar bone [7,9]. This is further supported by Jiang et al. [9], who noted that clear aligners induce less root resorption and bone dehiscence than fixed appliances, likely because of their removable nature and lower force magnitude.

The lack of significant differences in bone level changes between patients with and without interproximal stripping (both -0.89 mm) is noteworthy. Interproximal stripping, often used to address crowding, could theoretically exacerbate bone loss owing to reduced interseptal bone support. However, Darwiche et al. [12] reported no significant reduction in the inter-radicular bone after interproximal stripping with clear aligners, suggesting that careful execution of stripping within the recommended limits preserves bone integrity. This reinforces the safety of incorporating interproximal stripping into clear aligner protocols when clinically indicated. In the application of clear aligners, the practitioner is allocated the designated quantity of interproximal reduction, typically ranging from 0.2 to 0.5 mm for each contact point [13].

Class I and class II malocclusions demonstrated significant bone level changes (-0.90 mm and -0.86 mm, respectively), while class III changes (-0.34 mm) were non-significant (p = 0.078). This variation may be attributed to the biomechanical demands of different malocclusions. Class I and II malocclusions often involve anterior crowding or deep bite corrections, which require more pronounced tooth movements and may stimulate greater bone remodeling. In contrast, class III malocclusions in this study were less prevalent (4%) and typically involved minor spacing corrections, potentially explaining the reduced effect size. These findings are consistent with those of Baneshi et al. [14], who reported that clear aligners are effective for simple malocclusions treated using a non-extraction approach.

Multivariable linear regression analysis indicated that sex (p = 0.041) and treatment duration (p = 0.017) had small but significant effects on bone level changes, while other factors, such as age and malocclusion type, showed no significant associations (p > 0.05). The influence of sex may be linked to differences in bone density or hormonal factors affecting bone metabolism, as suggested by Choi et al. [15]. The effect of treatment duration aligns with the findings of Alfuriji et al. [16], who noted that longer treatment periods allow for sustained bone remodeling. However, the small effect sizes in the current study suggest that these factors may not be the primary drivers of bone-level changes, supporting the notion that clear aligner therapy produces relatively consistent periodontal outcomes in diverse patient profiles.

Compared to traditional fixed appliances, clear aligners appear to offer advantages in terms of periodontal health [9]. Fixed appliances are associated with higher risks of plaque accumulation, gingival inflammation, and bone loss owing to their non-removable nature and challenges in maintaining oral hygiene [17]. In contrast, the removability of clear aligners facilitates better oral hygiene as patients can brush and floss without obstruction. This is supported by Rossini et al. [7], who conducted a systematic review and found that clear aligners are associated with lower plaque indices and better periodontal health. The current study’s finding of minimal bone-level changes, particularly in periodontally healthy patients, further corroborates the periodontal safety of clear aligners.

However, it is critical to note that while clear aligners may reduce the risk of adverse periodontal outcomes, they do not eliminate them entirely. The significant bone level changes observed in this study, although small in magnitude, indicate that clinicians must remain vigilant in monitoring periodontal health throughout the treatment. Regular periodontal assessments, including probing depth and bleeding on probing, should be integrated into aligner therapy protocols to detect the early signs of bone loss or inflammation.

Clinical implications

The findings of this study highlight the need for meticulous treatment planning with clear aligner therapy owing to significant changes in alveolar bone levels, particularly in the mandibular regions. Orthodontists should use pre-treatment OPGs for baseline assessments and monitor periodontal health throughout therapy, especially in cases involving mandibular expansion or prolonged treatment. Thorough periodontal screening is essential for confirming patient eligibility and ensuring periodontal health. The pronounced bone changes in the mandibular anterior region suggest the optimization of force magnitudes and alignment sequencing to minimize periodontal stress. Patient education on maintaining excellent oral hygiene and adhering to 20 to 22 hours of daily aligner wear is critical to enhance outcomes and mitigate risks.

Limitations

The retrospective design limits control over confounding variables such as patient compliance with aligner wear or oral hygiene, potentially influencing bone-level changes. The sample size of 50 patients, while statistically justified, may not fully represent diverse malocclusions, particularly class III malocclusions, limiting generalizability. The OPGs used for measurements cannot capture three-dimensional bone changes, unlike cone beam computed tomography (CBCT), which is avoided because of radiation and cost concerns. This study’s focus on immediate post-treatment outcomes omits long-term stability data, and the lack of a control group (such as fixed appliances or untreated patients) hinders the attribution of changes solely to aligners. Moreover, due to the retrospective nature of the study, we could not do periodontal charting of the cases.

## Conclusions

This retrospective study concluded that clear aligner therapy resulted in statistically significant but clinically minimal changes in alveolar bone height, with more pronounced effects observed in the mandibular regions compared to the maxillary regions. The findings affirmed the periodontal safety of clear aligners for periodontally healthy adults, with consistent bone remodeling outcomes across various patient characteristics and treatment parameters, such as sex, malocclusion type, interproximal stripping, and treatment duration. The study highlighted the importance of meticulous treatment planning, including baseline and ongoing periodontal assessments, to ensure optimal outcomes. These results supported the use of clear aligners as a safe orthodontic treatment option, emphasizing the need for careful monitoring to maintain periodontal health throughout therapy.
